# Peritoneal dialysis vs. hemodialysis among patients with end-stage renal disease in Iran: which is more cost-effective?

**DOI:** 10.1186/s12882-024-03530-0

**Published:** 2024-03-06

**Authors:** Mahmood Yousefi, Satar Rezaei, Sakineh Hajebrahimi, Niloofar Falsafi, Fatemeh Keshvari-Shad

**Affiliations:** 1 National Center for Health Insurance Research, Tehran, Iran; 2 Department of Health Economics, School of Management and Medical Informatics, Tabriz University of Medical Sciences, Tabriz, Iran; 3 Research Center for Environmental Determinants of Health, Health Institute, Kermanshah University of Medical Sciences, Kermanshah, Iran; 4 Research Center for Evidence-Based Medicine, Iranian EBM Centre: A Joanna Briggs Institute (JBI) Center of Excellence, Tabriz University of Medical Sciences, Tabriz, Iran; 5 Farshchian Hospital, Hamadan University of Medical Sciences, Hamadan, Iran

**Keywords:** Cost-utility analysis, Quality adjusted life year, End-stage renal disease, Dialysis

## Abstract

**Background:**

There is little economic evidence on different modalities among patients with end-stage renal disease (ESRD) in Iran. This study aimed to assess the cost-utility of peritoneal dialysis (PD) and hemodialysis (HD) among ESRD patients in Iran.

**Methods:**

From the health system perspective and with a 10-year time horizon, we conducted a cost-utility analysis based on a Markov model to compare three strategies of PD and HD [the second scenario (30% PD, 70% HD), the third scenario (50% PD, 50% HD) and the fourth scenario (70% PD, 30% HD)] among ESRD patients with the current situation (PD, 3% vs. HD, 97%) as the basic scenario (the first scenario) in Iran. Cost data for PD, HD and kidney transplantation were extracted from the medical records of 720 patients in the Health Insurance Organization (HIO) database. The Iranian version of the EQ-5D-5 L questionnaire was filled out through direct interview with 518 patients with ESRD to obtain health utility values. Other variables such as transition probabilities and survival rates were extracted from the literature. To examine the uncertainty in all variables included in the study, a probabilistic sensitivity analysis (PSA) was performed. TreeAge Pro 2020 software was used for data analysis.

**Findings:**

: Our analysis indicated that the average 10-year costs associated with the first scenario (S1), the second scenario (S2), the third scenario (S3) and the fourth scenario (S4) were 4750.5, 4846.8, 4918.2, and 4989.6 million Iranian Rial (IRR), respectively. The corresponding average quality-adjusted life years (QALYs) per patient were 2.68, 2.72, 2.75 and 2.78, respectively. The ICER for S2, S3 and S4 scenarios was estimated at 2268.2, 2266.7 and 2266.7 per a QALY gained, respectively. The analysis showed that at a willingness-to-pay (WTP) threshold of 3,000,000,000 IRR (2.5 times the GDP per capita), the fourth scenario had a 63% probability of being cost-effective compared to the other scenarios.

**Conclusion:**

Our study demonstrated that the fourth scenario (70% PD vs. 30% HD) compared to the current situation (3% PD vs. 97% HD) among patients with ESKD is cost-effective at a threshold of 2.5 times the GDP per capita (US$4100 in 2022). Despite the high cost of PD, due to its greater effectiveness, it is recommended that policymakers pursue a strategy to increase the use of PD among ESRD patients.

## Introduction

The prevalence of chronic kidney disease (CKD), as one of the major health problems, is increasing globally and places a high financial and non-financial burden on patients, their families, the health system, and society as a whole [1–3]. A recent global study indicated that CKD is responsible for around 1.2 million deaths and is identified as the 12th leading cause of death worldwide [4]. In Iran, similar to other countries, the prevalence of CKD is high, and a systematic review study indicated the prevalence of CKD among the general population is 15.2% [5]. In addition to the significant burden of CKD, the quality of life of patients with CKD is lower than that of healthy people and some other chronic diseases as well [6, 7].

While drug interventions are highly effective in the early stages of kidney disease, patients in advanced stages require either kidney transplantation or dialysis. Of these two treatment options, kidney transplantation is superior, as it can reduce treatment costs and improve patients’ quality of life. As per existing literature [8–10], although kidney transplant is more cost-effective than dialysis for ESKD patients, the use of this strategy is not possible for all patients due to the shortage of organ sources, and annually few patients will have the chance to get a kidney transplant, so most ESKD patients should undergo dialysis.

However, in Iran, similar to many other countries, the use of HD is more prevalent [11, 12]. As per the latest report in Iran, by the end of 2015, the total number of patients with ESKD was about 58,000, of which 29,200 patients received HD, 1,624 received PD, and 27,000 patients received a kidney transplant [13]. There are several important points to note in Iran’s health system. First, the costs of PD are higher compared to HD. Second, the cost of dialysis services is fully paid by health insurance organizations. And finally, individuals have a greater tendency to use HD and kidney transplantation instead of PD. Therefore, optimal management of resources and proper planning for these patients are very important for health policymakers.

Although many studies have been conducted regarding the quality of life of HD, PD and renal transplantation patients [14, 15] and the costs of dialysis modalities [16, 17] in Iran, there is little information on the cost and effectiveness of HD and PD among patients with ESKD. To fill this gap in the literature, the current study was conducted to compare the cost-utility of HD and PD among ESKD patients in Iran. The evidence provided in the study can help policymakers optimize the management of scarce resources in the health system, design cost-effective interventions with priority given to low-cost interventions with high effectiveness, and finally, properly respond to patients’ demands in Iran and similar settings.

## Method and materials

A full economic evaluation, cost-utility analysis, was done to compare two dialysis modalities - HD and PD - among ESRD patients in Iran from the health system perspective. To conduct a proper economic evaluation study, we followed the various steps as per the reference guidelines of the National Institute for Health and Care Excellence (NICE) [18]. The costs and outcomes were estimated based on a 10-year timeframe and health system perspective. Based on this perspective, we included the costs of equipment, facilities, supplies, medications, and human resources associated with providing each dialysis modality. We excluded indirect costs and direct non-medical costs borne by patients and caregivers, such as travel expenses and productivity losses. With regard to effectiveness, the health system perspective does not affect the effectiveness of the study. Specifically, the effectiveness outcomes of mortality, hospitalization rates, and quality-adjusted life years are clinical and patient-centered results that are independent of the perspective taken [19, 20].

**Fig. 1:**
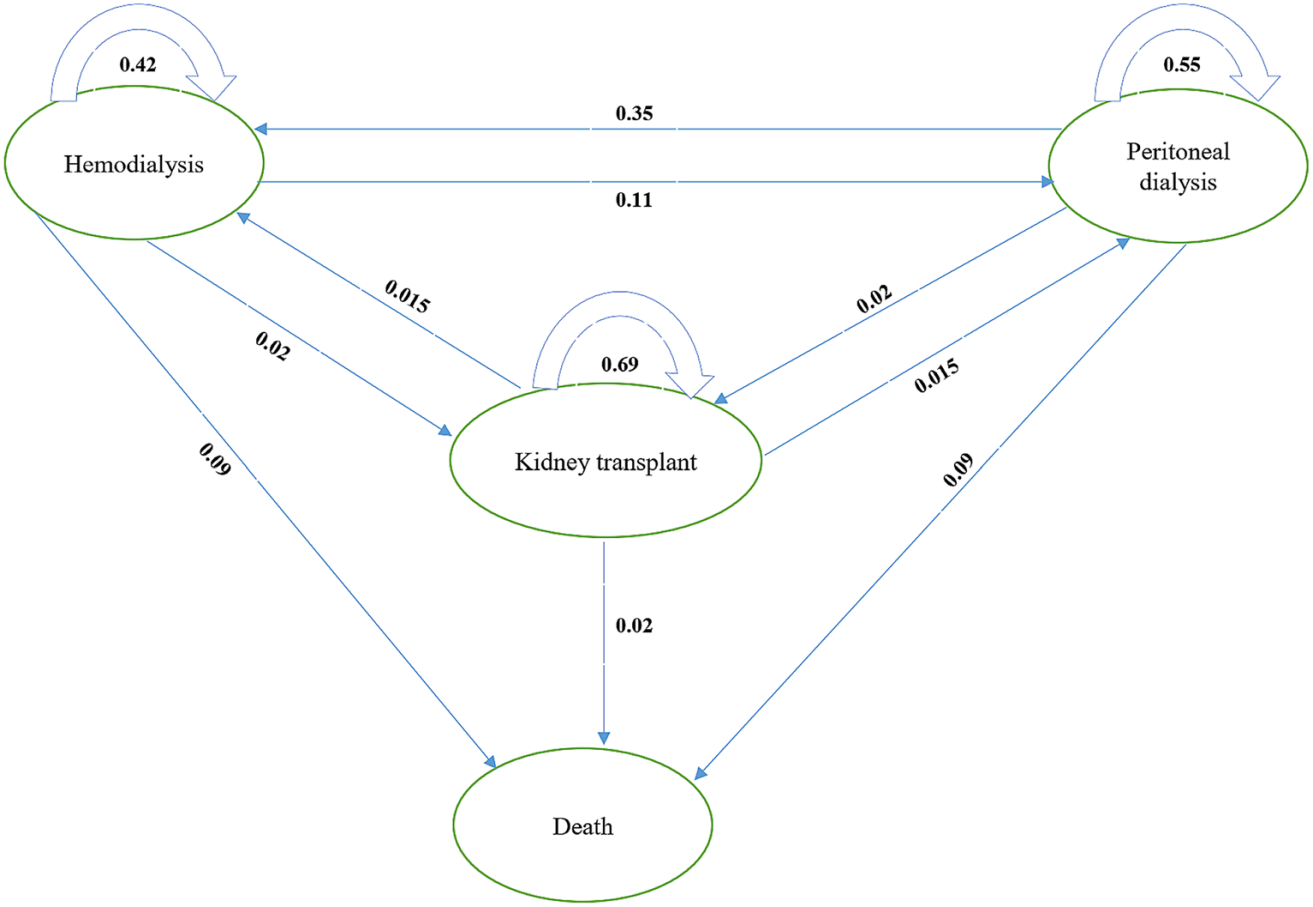
Markov model for CEA of HD vs. PD

The economic model was constructed according to the nature of the disease, literature review, the process of performing dialysis modalities, state transition probabilities, clinical outcomes in terms of QALY, and patient costs. The simple diagram of the Markov model used in the study with a one-year cycle length and 10-year timeframe is illustrated in Fig. 1. Four health states were considered, including HD, PD, kidney transplant, and death. The survival and mortality rates for HD and PD were obtained from a national cohort study [21], and the survival rate for kidney transplant was extracted from a meta-analysis study [22].

The probability of transition from PD and HD to kidney transplantation was obtained from the health insurance database. Also, the probability of rejecting the kidney transplant and returning to the PD state was extracted from a cost-utility study [23]. The probability of patients transitioning to the two states of HD and PD after transplant rejection was assumed to be the same.

In this study, four scenarios were examined. The base scenario was defined based on available data (rate of PD in ESKD patients), which was 3% for PD compared to 97% for HD. These figures were extracted from the health insurance information. The other scenarios were determined assuming an increase in PD patients compared to HD: the second scenario (30% PD, 70% HD), the third scenario (50% PD, 50% HD), and the fourth scenario (70% PD, 30% HD).

To estimate the costs of dialysis modalities, first, the list of HD, PD, and kidney transplant patients covered by the Iranian Health Insurance Organization (IHIO) was obtained from the database of the National Institute for Health Insurance Research in 2022. The healthcare system in Iran relies on several health insurance funds to provide coverage for the entire population as mandated by law. The largest of these is the IHIO, which is legally required to cover over half of the country’s citizens. Eligibility for coverage under this organization is defined by legislation, and enrollment is compulsory for most people meeting the criteria. Once enrolled, there is little flexibility to change funds or insurers. The system is structured so that each eligible citizen must remain in their assigned fund based on the guidelines. This means that for the majority enrolled in the IHIO, they cannot opt out or select alternate coverage even if desired. Next, according to the number of patients with CKD in seven provinces (Tehran, Yazd, Fars, West Azarbaijan, East Azarbaijan, Hamadan, and Qazvin) and the total population of the provinces, 760 patients were selected as the final sample. The average age was 57.7 years, with a standard deviation of 16.6. The sample contained more males (60%) than females (40%). The largest proportion of patients were on HD (43.6%), followed by those with a kidney transplant (42.6%), and PD (13.8%). The average annual direct medical costs per patient, including medications, physician visits, lab tests, imaging services, dialysis service, and hospitalization, were estimated. The total direct medical costs of PD, HD, kidney transplant in the first year, and kidney transplant in the second year were 1,143,654,799 IRR, 848,855,549 IRR, 538,750,671 IRR, and 64,458,254 IRR, respectively. All cost data were calculated according to 2022–2023 prices. According to the NICE guidelines [18], the Iranian version of the EQ-5D-5 L questionnaire [24] was used through direct interviews with 518 patients to extract the utility values for patients with PD (*n* = 76), HD (*n* = 312), and kidney transplantation (*n* = 130). The questionnaire includes 5 dimensions (mobility, self-care, usual activities, pain/discomfort, and anxiety/depression), and each dimension has 5 levels (no problems, slight problems, moderate problems, severe problems, and extreme problems). The mean (SD) utility values were 0.550 (0.468) for PD patients, 0.423 (0.549) for HD patients, and 0.695 (0.341) for kidney transplant patients.

### Inclusion and exclusion criteria

The inclusion criteria for the study comprised all patients with ESKD who were treated with PD or HD and covered by the Health Insurance Organization in the selected provinces, were at least 18 years and above, and finally for whom at least three months had passed since the start of their treatment with either PD or HD.

### Cost-effectiveness analysis

The incremental cost-effectiveness ratio (ICER) was used to determine the most cost-effective scenarios as follow:

$$ ICER= \frac{{C}_{2}-{C}_{1}}{{E}_{2}-{E}_{1}}$$

Where C shows the costs and E shows the effectiveness.

A willingness-to-pay threshold is needed to analyze cost-utility results. In developing countries, the most commonly used threshold is the one recommended by the WHO, which is calculated based on GDP per capita [25]. According to this recommendation, if the ICER for a healthcare intervention in a country is less than the GDP per capita, that intervention is chosen as very cost-effective. In addition, if the ICER falls between 1 and 3 times the GDP per capita, the intervention is considered cost-effective. Finally, interventions with an ICER more than 3 times the GDP per capita are identified as not cost-effective. In this study, we used the WHO recommendation and the GDP per capita was $4,100 US at the time of this study. In 2022, the GDP per capita for Iran was equal to US $4,100 according to International Monetary Fund (IMF) data [26]. In 2022, US$1 was almost equal to 3,000,000 IRR [27].

### Sensitivity analysis

Considering uncertainty regarding parameters included in the model, including utility values, costs, and probability of transition within and between states, a probabilistic sensitivity analysis using Monte Carlo simulation with 1,000 repetitions was carried out. A gamma distribution for cost data and a beta distribution for other variables such as transition probabilities and utility values were considered. All data analyses were done through TreeAge software 2020.

## Results

**Fig. 2:**
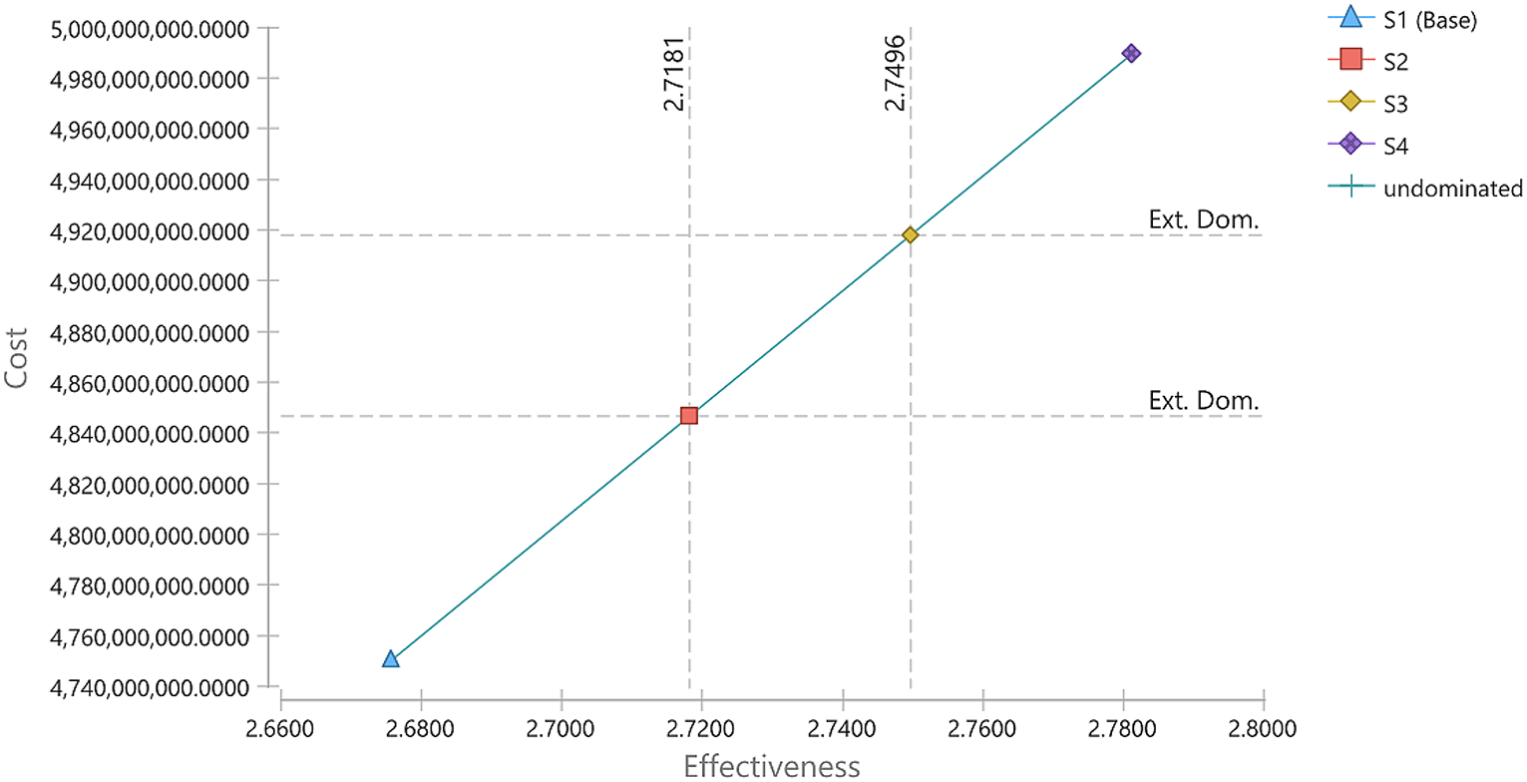
Cost-effectiveness analysis of different modalities of PD vs. HD

The results of the cost-utility analysis of different strategies of HD and PD are presented in the Table 1. As indicated in Table 1, the average 10-years costs associated with S1, S2, S3 and S4 were 4750.5, 4846.8, 4918.2, and 4989.6 million Iranian Rial (IRR), respectively. Corresponding average QALY per patients were 2.68, 2.72, 2.75 and 2.78, respectively. The ICER for S2, S3 and S4 scenarios was estimated at 2268.2, 2266.7 and 2266.7 per QALY gained, respectively. The obtained ICER for three strategies indicated that all of them at threshold considered in the study (3000 million IRR) are cost-effective. The results of cost-utility analysis plane are illustrated in Fig. 2. It is clear from the figure that all four scenarios are located in region one where both cost and effectiveness are high. The fourth scenario is chosen as cost-effective scenario due to its higher effectiveness despite the higher cost compared to the second and third scenarios.

**Table 1:** Results of the cost-utility analysis of different strategies of HD and PD

Scenario	Costs (million IRR)	QALY	Incremental costs	Incremental QALY	ICER
S1 (base case)	4750.5	2.6756	-	-	-
S2	4846.8	2.7181	96.4	0.0425	2268.2
S3	4918.2	2.7496	71.4	0.0315	2266.7
S4	4989.6	2.7811	71.4	0.0315	2266.7

**Fig. 3:**
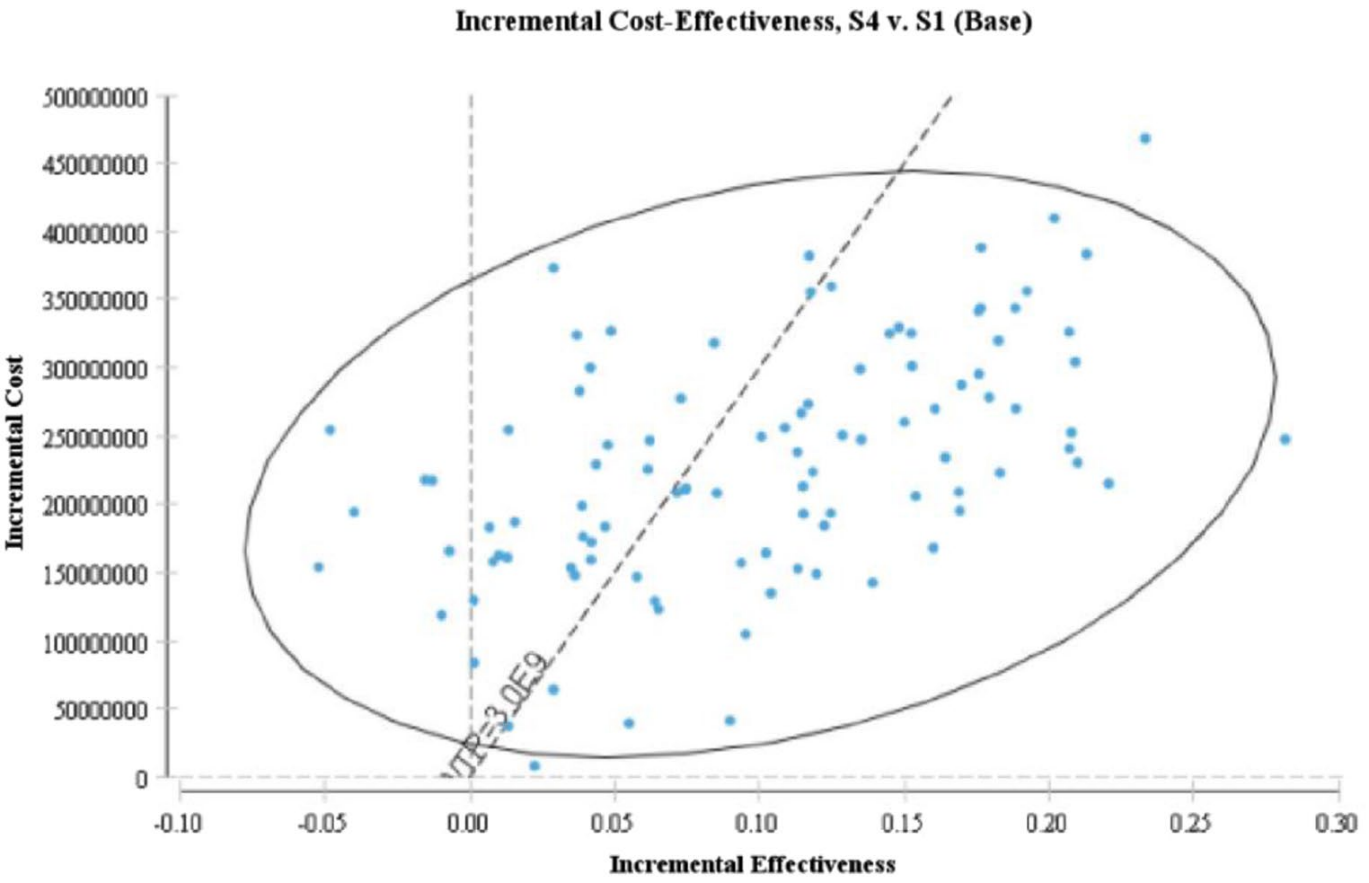
Incremental cost-effectiveness scatterplot of PD and HD among ESRD patients

In order to examine the uncertainty from all variables related to costs and outcomes included in the model, probabilistic sensitivity analysis (PSA) using Monte Carlo simulation with 1000 iterations was performed (Fig. 3). The results showed that in 65% of the simulations, the fourth scenario (70% PD vs. 30% HD) was the dominant scenario, whereas the base scenario (the first scenario) (3% PD vs. 97% HD) was dominant in 35% of the simulations.

**Fig. 4:**
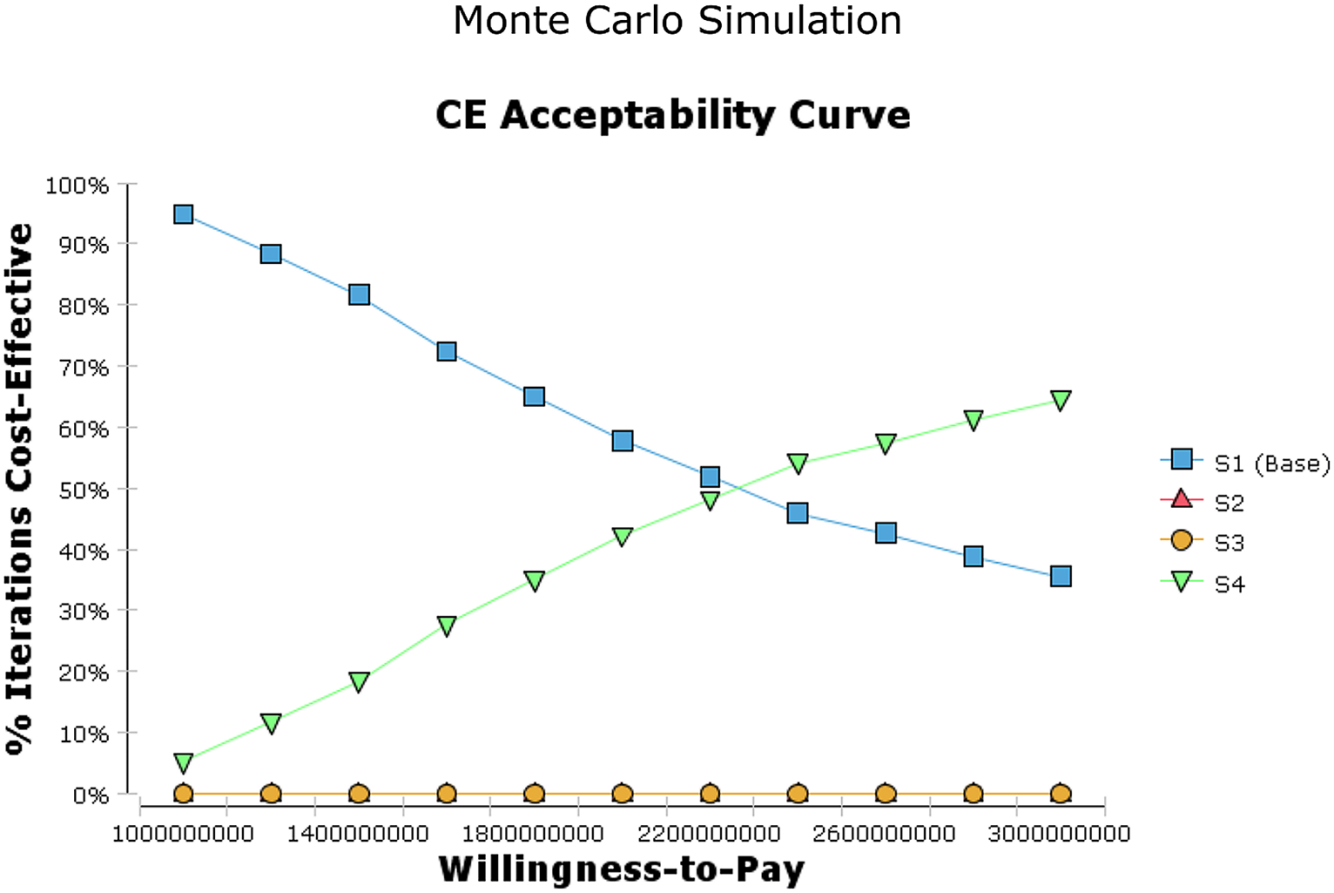
Cost effectiveness acceptability curve (CEAC) for four strategies of PD and HD

The cost-effectiveness acceptability curve (CEAC) presents the probability of the strategies 2, 3 and 4 of PD and HD being cost-effective compared to the base scenario (the first scenario) at the different levels of willingness to pay (WTP) thresholds (Fig. 4). The analysis showed that considering a discount rate 3.5%, at a WTP of 2,300,000,000 IRR (1.9 times the GDP per capita), the base scenario had 50% probability of being cost-effective. At a WTP threshold 3,000,000,000 IRR (2.5 times the GDP per capita), probability of being cost-effective of the fourth scenario was about 63%.

## Discussion

Chronic kidney disease substantially impacts patients, health systems, and society through treatment costs, quality of life, and healthcare spending. This study assessed the cost-utility of PD versus HD for end-stage kidney disease patients in Iran from a health system perspective. The results showed that the fourth scenario (70% PD and 30% HD) is cost-effective compared to current practice for ESRD patients. The ICER for the three scenarios (second, third, and fourth) compared to current practice was 2268.2, 2266.7 and 2266.7 million IRR per QALY gained, respectively. Therefore, the fourth scenario is chosen as the preferred option due to greater effectiveness compared to the second and third scenarios. This intervention is not very cost-effective but since it lies within the selected WTP threshold, it is introduced as a cost-effective scenario.

In a study conducted in China [28], three different scenarios were compared to the current situation (HD 73%; PD 14%; kidney transplant 13%). The second scenario was HD 47%; PD 40%; transplant 13%. The third scenario was HD 52%; PD 14%; transplant 34%. The fourth scenario was HD 26%; PD 40%; transplant 34%. That study indicated PD was cost-effective compared to HD in a 5-year time horizon. Additionally, kidney transplantation was cost-effective compared to PD at a WTP threshold of US$44,300. They concluded kidney transplantation is the most cost-effective strategy, followed by PD and HD. In another study in Thailand, PD was found to be more cost-effective compared to HD based on the current WTP threshold [29]. In a study by Putri et al. in Indonesia [30], PD provided good value for money versus HD for ESRD patients based on cost analysis and QALYs gained. Our study indicated the highest and lowest utility values were for kidney transplantation and HD, respectively. The utility values for transplantation, HD and PD were 0.70, 0.42, and 0.50, consistent with other studies [31–33]. The highest direct medical costs were for PD. As the largest purchaser of health services in Iran, the Health Insurance Organization should take steps towards strategic purchasing and optimal management of dialysis resources given the high costs imposed by these services.

Although this is the first national study on cost-utility of PD and HD for ESRD in Iran, there are some limitations. We used some transition probabilities from non-Iranian studies, although we did probabilistic sensitivity analysis to address this. Secondly, we used a health system perspective and did not include all costs such as productivity and caregiver costs. Future studies could compare cost-effectiveness from a societal perspective.

## Conclusion

The study demonstrated that from Iran’s health system perspective and 10-year time horizon, the fourth scenario (70% PD vs. 30% HD) compared to the current situation (3% PD vs. 97% HD) among patients with ESKD is cost-effective at a threshold between 1 and 3 times Iran’s GDP per capita. Despite the higher direct medical costs of PD, due to its greater effectiveness, it is recommended that policymakers pursue a strategy to increase the use of PD among ESRD patients. At the same time, actions should be taken to increase bargaining power and reduce the price of interventions, so that the cost per QALY falls within a more acceptable range closer to the lower end of the threshold.

## Data Availability

Since this study utilized secondary data from hospital records and previous studies, informed consent was not required. The corresponding author can make the data available upon reasonable request.
